# Pott’s puffy tumor: a need for interdisciplinary diagnosis and treatment

**DOI:** 10.1007/s00106-021-01134-w

**Published:** 2022-01-24

**Authors:** Jan Philipp Kühn, Stefan Linsler, Nasenien Nourkami-Tutdibi, Sascha Meyer, Sören L. Becker, Umut Yilmaz, Bernhard Schick, Alessandro Bozzato, Philipp Kulas

**Affiliations:** 1 Hospital of Otorhinolaryngology, Head and Neck Surgery, Saarland University Medical Center, Homburg, Germany; 2 Hospital for Neurosurgery, Saarland University Medical Center, Homburg/Saar, Germany; 3 Hospital for General Pediatrics and Neonatology, Saarland University Medical Center, Homburg/Saar, Germany; 4 Institute of Medical Microbiology and Hygiene, Center for Infectious Diseases, Saarland University Medical Center, Homburg/Saar, Germany; 5 Hospital for Neuroradiology, Saarland University Medical Center, Homburg/Saar, Germany

**Keywords:** Osteomyelitis, Frontal bone, Frontal subperiosteal abscess, Children, Adolescents

## Abstract

Pott’s puffy tumor (PPT) is an infection of the frontal sinus with subperiosteal and intracranial abscess formation and one of the rare entities in pediatrics. We present a series of four cases of PPT that occurred in two children (6 and 9 years) and in two young adults (17 and 19 years). All patients were treated by an interdisciplinary team of pediatric, neurosurgical, ENT, radiological, and neuroradiological specialists. Antibiotic treatment was combined with single endoscopic surgery in one case and combined endoscopic sinus surgery with an open transcranial approach to drain intracranial abscess formation in three cases. It is important to be aware that PPT occurs in children with the finding of intracranial abscess formation. Therefore, a close interdisciplinary cooperation for successful treatment is needed in this rare disease.

## Introduction

Pott’s puffy tumor (PPT) is a rare phenomenon, characterized by localized forehead swelling, which was first described by Sir Percival Pott in the eighteenth century as an abscess formation and extradural empyema in relation to frontal head trauma [7]. Most frequently, PPT occurs after untreated or inadequately treated sinusitis [2, 10]. As a complication of the latter, it can lead to osteomyelitis by spreading through the frontal bone [4]. Most bacteria found in PPT correspond to community-acquired sinusitis such as *Streptococcus* spp., *Staphylococcus* spp., *Haemophilus influenzae, Klebsiella* spp., anaerobes and enterococci, with staphylococci being the most common agents [2, 3]. Of note, since many of these bacteria are also commensals of the skin, it is frequently challenging to estimate their actual clinical relevance when these bacteria are detected in microbiological analyses of swabs or tissue samples. Pott’s puffy tumor can be found in all age groups. It predominantly occurs in adolescents with a developed frontal sinus due to a higher incidence of upper respiratory tract infections and an increased risk of acute bacterial sinusitis [9]. Most cases are previously healthy patients, and sometimes PPT can occur as a result of cocaine abuse, in the context of dental infection, or as a late complication after neurosurgical interventions [3].

Frontal sinuses are absent at birth, begin to be pneumatized by 2 years of age, and are almost fully developed in adolescence. Venous drainage originates through diploic veins that communicate with the dural venous sinuses possibly contributing to septic emboli [9]. Intracranial complications such as cavernous sinus and dural venous sinus thrombosis, meningitis, epidural, subdural, or intraparenchymal abscess appear due to venous drainage or direct extension. The most common symptoms are purulent rhinorrhea, headache, periorbital swelling, fever, vomiting, and other signs of meningitis or encephalitis [11, 17]. Computed tomography (CT) is used to plan treatment and magnetic resonance imaging (MRI) is of paramount importance in identifying intracranial complications [12]. Although PPT is usually an indication for emergency surgical therapy, a purely conservative therapy can be considered in selected cases.

Here, the authors present the cases of several patients with PPT and their treatment seen at a university medical center in southwest Germany in 2019.

## Case reports

### Patient 1

A 6-year-old child was referred to our clinic from the pediatric department hospital with an unclear swelling of the forehead. The patient has already been treated in hospital with ampicillin/sulbactam for frontal sinusitis accompanied by fever for 6 days. After discharge, the boy presented with a discrete swelling of the forehead and was readmitted to hospital.

On physical examination, there were no additional symptoms apart of the non-tender painless forehead swelling. At readmission, fever was absent, but the parents described recurrent fever episodes over several weeks as well as a recurrent sinusitis, which had been treated over a long period with different antibiotics. Laboratory results showed an elevated C‑reactive protein (CRP) level of 61 mg/l (normal range: 0.0–5.0 mg/l) and white blood cells (WBC) of 20.6 (normal range: 4.8–12.0).

**Fig. 1:**
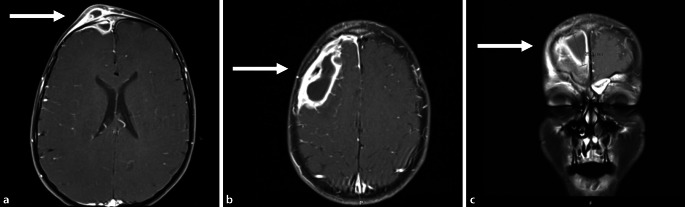
Subtotal padding of the not yet fully developed frontal sinus and a frontobasal epidural (**a** coronal) as well as an intradural abscess formation (**b** axial and **c** coronal) (*arrows*)

Ultrasound revealed a blurred clearly hypoechoic mass with thickening of the overlying tissue toward the skin. As far as it was detectable, the underlying bone was intact without any interruption of the corticalis intracranially. On suspicion of PPT, MRI and CT studies were initiated. The MRI examination revealed osteomyelitis of the right frontal bone with accompanying subperiosteal and subdural abscesses causing a local space-occupying effect and beginning meningoencephalitis. Furthermore, frontal sinusitis was described as the cause of the disorder and in the synopsis of all the findings, PPT was assumed (Fig. 1).

The patient underwent immediate surgery with frontolateral craniotomy. The destructed (small/less pneumatized) frontal sinus was opened to the ethmoid cells, with evacuation and drainage of the cranial, epidural, and subdural abscess. On endonasal inspection, no remnant abscess formations were found in the sinonasal cavities.

After successful drainage, the further course was uncomplicated. A short post-ventilation phase followed and the patient could be extubated without any problems. Microbiological cultures grew *Streptococcus intermedius*, and the antibiotic treatment was switched to cefotaxime and clindamycin for 14 days. The patient was discharged home in good condition without neurological sequelae.

### Patient 2

A 19-year-old male patient presented to our clinic with an acute right orbital and forehead swelling. He had recently undergone an *alio loco* endonasal operation because of similar symptoms 20 days earlier. In this case, the diagnosis had been a right-sided frontal sinus pyocele with an orbital complication. Therefore, functional endoscopic sinus surgery (FESS) had been performed to relieve the pyocele.

**Fig. 2:**
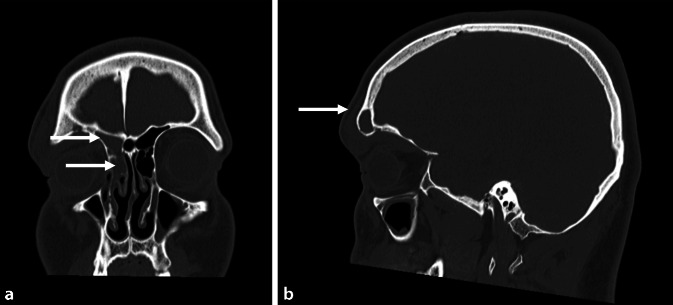
Ethmoid and frontal sinusitis on the right side as well as an infraorbital abscess with a connection to the frontal sinus (*arrows*) (**a** coronal and **b** sagittal)

An additional ophthalmologic examination showed no pathological findings and no signs of visual impairment. Fever and pain were absent and laboratory chemical analysis showed a CRP level of 9.6 mg/l and a WBC of 9.9. Osteomyelitis was revealed at the CT examination as a low-grade frontal erosion of the bony skull base (Fig. 2). Conservative treatment was initiated with high-dose intravenous antibiotic therapy with ceftriaxone 4 g–0–4 g. Under this treatment, the symptoms improved significantly, and he was discharged from hospital after 4 days.

The patient presented with progressive symptoms and a recurrent swelling of the right orbit and forehead to our clinic 8 days later. Revision surgery was performed with transnasal frontal sinus surgery type IIc and orbital abscess splitting. On microbial cultures, *S. intermedius* was detected and the antibiotic therapy was continued with ceftriaxone. After 5 days, the patient could be discharged in good condition with unrestricted vision and without any recurrence to date.

### Patient 3

**Fig. 3:**
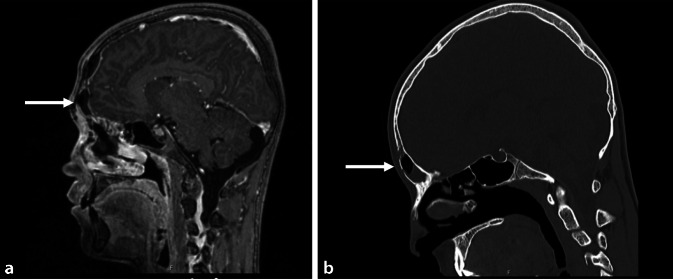
Pott’s puffy tumor with a connection to the frontal sinus of the left side (*arrows*) (**a** MRI-, **b** CT-scan, sagittal)

A 17-year-old male patient presented to our hospital with a history of fever, cough, and cephalgia for 2 days. The initial treatment was symptom-based with a mainly analgesic and antipyretic therapy. Due to a swelling of the forehead, the patient was referred to the clinic for otorhinolaryngology. Laboratory results revealed a highly elevated CRP level of 273.3 and a WBC of 15. On suspicion of PPT, MRI and CT examinations were initiated and confirmed the diagnosis of an acute pansinusitis. The CT scan additionally revealed two small encapsulated epidural abscesses in the frontal paramedic area with a connection to the left frontal sinus: 8 × 9 mm and 14 × 9 mm (Fig. 3). An intracerebral abscess could be excluded. The patient underwent immediate surgery of the frontal sinus. A transnasal type I drain according to Draf in combination with an endoscopic sinus surgery and an abscess relief through a cut in the corrugator fold was performed. Cranialization of the frontal sinus was also discussed but was postponed initially.

The patient received antibiotic therapy with ceftriaxone. On follow-up MRI and CT studies, no regression of the described epidural formations was seen, so that finally cranialization of the frontal sinus and evacuation of the epidural abscess were performed. The surgery was made as indicated, similar to the first case described here. The patient recovered without any neurological sequalae and was discharged in good general condition under further coverage with metronidazole and ceftriaxone for 2 weeks.

### Patient 4

A 9-year-old girl presented to hospital with persistent and progressive cold symptoms for about 6 weeks. Upfront antibiotic treatment did not lead to an improvement of her symptoms and her overall condition.

**Fig. 4:**
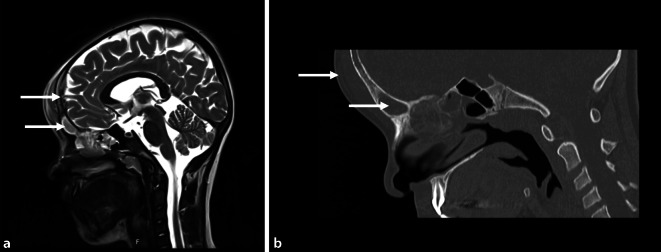
Frontal Pott’s puffy tumor with epidural empyema and frontal/ethmoid sinusitis (*arrows*) (**a** MRI-, **b** CT-scan, sagittal)

On initial presentation, the patient’s parents described recurring episodes of fever of up to 40 °C and a newly developed forehead swelling. Laboratory results showed a slightly elevated CRP level of 30.3 mg/l and a WBC of 10.1. Both the MRI and CT studies revealed sinusitis with frontal osteomyelitis accompanied by an epidural and subgaleal abscess formation without intracranial involvement (Fig. 4). The patient underwent immediate surgery. In a combined ENT/neurosurgical intervention, endoscopic sinus surgery on the left side was performed followed by and an osteoplastic craniotomy with removal of the epidural abscess as well as a drilling out of the osteomyelitis duct. Antibiotic therapy with cefotaxime and clindamycin was initially started and switched to ampicillin/sulbactam after detection of *S. intermedius*. Antibiotic treatment was administered for 14 days. The epidural abscess formation and the soft tissue swelling completely resolved clinically and on follow-up imaging. The patient could be discharged home without neurological sequalae.

## Discussion

### Incidence of age

As described earlier, most cases of PPT occur during adolescence, most likely facilitated by developmental circumstances [9, 11]. The diameter and the flow rate in diploic veins that drain the frontal sinuses increases in adolescence, which promotes hematogenous spread of infections into the bone and intracranially [6, 21]. Additionally, the skull bone that limits the frontal sinus is much thinner in adolescents than in adults, and thus there is a shorter path between the infection and the bone contributing to a hematogenous spread of infections. Finally, pneumatization of the frontal sinuses is not completed before the age of 14–15 years [12, 19]. When the paranasal sinus system develops, the frontal sinuses grow from the ethmoid air cells continuously in a cranial direction. This explains the fact that small children are less likely to be affected by PPT due to an incomplete or less pneumatized frontal sinuses. Our cases, however, show that even younger children at the age of 6 and 9 years can develop a corresponding symptomatology (Table 1). Although the sinuses are not yet fully formed, infections of the anterior ethmoid can find their way across the frontal recess to the area of the later frontal sinus.

**Table 1:** Bacterial pathogens and the antibiotic as well as surgical treatment

Patient	Bacterial pathogens	Antibiotic therapy	Treatment
**Patient 1** **age 6 ♂**	*Streptococcus intermedius*	Sultamicillin and clindamycin	Frontolateral craniotomy and FESS
**Patient 2** **age 19 ♂**	*Streptococcus intermedius*	Ceftriaxone	FESS in combination with an external approach to the orbit
**Patient 3** **age 17 ♂**	*Eikenella corrodens, Staphylococcus capitis*	Metronidazole and ceftriaxone	FESS in combination with an external approach to the frontal sinus
**Patient 4** **age 9 ♀**	*Streptococcus intermedius, Corynebacterium* spp.	Ampicillin/sulbactam	Frontolateral craniotomy in combination with FESS

*FESS* functional endoscopic sinus surgery

### Pathogenesis

In ethmoidal or frontal sinusitis, the infection spreads via the diploic veins or through the thinnest bone, in case of the frontal sinus, it is via the floor and the frontal wall. If the infection spreads into the bone, it leads to demineralization with necrosis, resulting in osteomyelitis [23]. An infection can spread via retrograde thrombophlebitis; in the case of destruction of the anterior wall of the frontal sinus, a subperiosteal abscess can be formed and by destruction of the posterior wall an epidural abscess can be formed. If the inferior wall of the sinus is affected, the infection can spread to the orbit and form the clinical picture of an orbital complication with preseptal cellulitis up to an orbital abscess. The spread of the infection is further promoted by the lack of venous valves in the diploic veins.

### Diagnosis

The diagnosis of PPT is made on the basis of a clinical examination. Most patients show a frontal swelling in the forehead area partially reaching the orbit. Many patients experience frontal headache, clear or purulent rhinorrhea, and/or fever [2, 8]. The differential diagnosis of such a forehead swelling includes, beside acute sinusitis, an infected atheroma, a carbuncle, or a condition after a head trauma with hematoma [12, 20]. The blood count is in many cases increased WBC as well as an elevated erythrocyte sedimentation rate and CRP level [1]. The medical history of a head trauma or acute and chronic sinusitis may be helpful for the diagnosis. If there is intracranial involvement, patients may also show signs of increased intracranial pressure indicated by nausea, focal or neurological deficits, and even loss of consciousness [2, 15]. An ultrasound examination can more precisely differentiate the entity of the forehead swelling. Here, liquid components such as pus in the swelling can be revealed and structures of the frontal bone can be assessed more precisely [16]. As a less invasive method, ultrasound should be favored especially for children instead of MRI and/or CT [18]. To identify the destruction of the frontal bone and subperiosteal fluid collection, contrast-enhanced CT is considered most effective [5]. In addition, CT is crucial for upfront surgical intervention, indicating whether other paranasal sinuses in addition to the frontal sinus are affected or if there is involvement of the orbits [11, 14]. If there is any suspicion of intracranial and/or intracerebral involvement, MRI should be performed in time to prove the necessity of intracranial interventional treatments. In addition to the meninges, the intraorbital structures can be evaluated and a sinus vein thrombosis may be excluded or confirmed [11]. To plan the procedure further, especially with regard to a transnasal drainage of the focus in the sense of FESS, a CT examination should always be performed to carry out a navigated endonasal sinus surgery and to reduce the intraoperative risk.

### Microbiology

Since in the majority of cases PPT is associated with an upper respiratory tract infection, Streptococcus (alpha- and beta hemolytic streptococci), *Haemophilus influenzae, Staphylococcus aureus* and other anaerobes (Fusobacterium and Bacteroides species) play a crucial role [12]. In our cases, *Streptococcus intermedius* was found in three of four patients, *Staphylococcus capitis* was detected in one (Table 1). *Streptococcus intermedius* is known for its high pathogenic potential and is reported to be associated with intracranial abscesses in patients with concurrent sinusitis and patients with multiple risk factors [22]. In contrast to this, all our patients were young, healthy, and immunocompetent. Early and calculated antibiotic therapy makes the complication of acute sinusitis ever rarer and a prolonged antibiotic therapy should be administered after resistance testing, especially in difficult anatomical conditions and up to 6 weeks after the surgical intervention [22]. The antibiotic susceptibility testing of *Streptococcus intermedius* showed an antimicrobial susceptibility against penicillin, ampicillin, cefotaxime, ceftriaxone, and vancomycin. For *Staphylococcus capitis,* antibiotic sensitivity to flucloxacillin, vancomycin, ciprofloxacin, doxycycline, and co-trimoxazole was found.

### Treatment

The gold standard in the therapy of PPT is calculated antibiotic therapy in combination with an operative drainage of the infection. A rapid and effective treatment is of crucial importance to prevent further complications such as an epidural or intracerebral complication. In this case, the antibiotic treatment should already be started when there is clinical suspicion of PPT and should be adapted to the corresponding pathogen spectrum after obtaining microbiological material. Initial treatment should be commenced by a broad-spectrum antibiotic with good blood–brain barrier passage, such as ceftriaxone, which was used in our cases [14]. Nonetheless, the key pillar of therapy is surgical drainage of the abscess. The approach to should be chosen depends on the individual anatomy and extent of the infection. There is the possibility of an endonasal approach and even an open frontal sinus access or the combination of both. Intracranial abscess formation needs additional neurosurgical treatment [12]. Here, external drainage has the distinct advantage that the entire area of the frontal sinus can be overseen and the bone that is affected by osteomyelitis can be removed [11]. In many cases, frontal sinus trepanation represents the access of choice [4]. By contrast, FESS is a minimally invasive technique and represents the possibility of tissue-preserving drainage. Here, in contrast to an open external access, the osteomeatal complex, which is the narrowest point of drainage of the frontal sinus, can be viewed and treated. In addition to the advantage of lacking external scars, patients after FESS, in contrast to an open approach, have a significantly shortened convalescence and thus a much shorter hospitalization time. Only single drainage of the subperiosteal abscess also appears to be associated with an increased recurrence rate or further complications [11, 13]. According to these findings, surgical therapy and rehabilitation should be carried out in close cooperation between the participating disciplines of ENT, neurosurgery, radiology, ophthalmology, microbiology/infectious diseases, and pediatrics. Sole antibiotic therapy without any operative treatment seems to be associated with a high rate of recurrences and—according to the current state of research—cannot replace surgical therapy [16]. In the four cases described here, drainage of the frontal sinus was solely endonasal in two cases whereas the other two patients had a craniotomy in combination with external access for the treatment of the subgaleal and epidural as well as intradural abscess formations (Table 1).

## Practical conclusion

- Pott’s puffy tumor still remains a rare complication of acute or chronic sinusitis, especially in young children.
- The consistent treatment of sinusitis, even in infancy without a fully pneumatized paranasal sinus system to prevent intracranial complications, is of crucial importance.
- The literature as well as the cases described here show that even in infancy, acute sinusitis can lead to life-threatening complications.
- Our cases underscore that Pott’s puffy tumor always requires close interdisciplinary collaboration to avoid long-term complications and to support a complete rehabilitation.

## Declarations

For this article no studies with human participants or animals were performed by any of the authors. All data collection was in accordance with the ethical standards indicated in each case.

The supplement containing this article is not sponsored by industry.
